# An attentional residual feature fusion mechanism for sheep face recognition

**DOI:** 10.1038/s41598-023-43580-2

**Published:** 2023-10-10

**Authors:** Yue Pang, Wenbo Yu, Yongan Zhang, Chuanzhong Xuan, Pei Wu

**Affiliations:** 1 College of Mechanical and Electrical Engineering, Inner Mongolia Agricultural University, Hohhot, 010018 China; 2 College of Computer and Information Engineering, Inner Mongolia Agricultural University, Hohhot, 010018 China

**Keywords:** Biological techniques, Computational biology and bioinformatics, Zoology

## Abstract

In the era of globalization and digitization of livestock markets, sheep are considered an essential source of food production worldwide. However, sheep behavior monitoring, disease prevention, and precise management pose urgent challenges in the development of smart ranches. To address these problems, individual identification of sheep has become an increasingly viable solution. Despite the benefits of traditional sheep individual identification methods, such as accurate tracking and record-keeping, they are labor-intensive and inefficient. Popular convolutional neural networks (CNNs) are unable to extract features for specific problems, further complicating the issue. To overcome these limitations, an **Attention Residual Module (ARM)** is proposed to aggregate the feature mapping between different layers of the CNN. This approach enables the general model of the CNN to be more adaptable to task-specific feature extraction. Additionally, a targeted sheep face recognition dataset containing 4490 images of 38 individual sheep has been constructed. Furthermore, the experimental data was expanded using image enhancement techniques such as rotation and panning. The results of the experiments indicate that the accuracy of the VGG16, GoogLeNet, and ResNet50 networks with the ARM improved by 10.2%, 6.65%, and 4.38%, respectively, compared to these recognition networks without the ARM. Therefore, the proposed method for specific sheep face recognition tasks has been proven effective.

## Introduction

The globalization of the livestock market has driven the rapid expansion of large-scale sheep farming to meet the increasing demand for high-quality agricultural and livestock products, including mutton, cashmere, and sheep milk. However, this growth has brought forth numerous challenges, which necessitate prompt attention and innovative solutions. One of the most significant problems facing the industry is the increased disease risk in individual sheep due to the high-bed confinement and off-site short-term fattening farming model^1^. To reduce this risk and improve breeding efficiency, the insurance industry has entered the livestock sector, offering individual or group insurance to protect the interests of herders and promote the industry’s sustainable development^2^, However, fraudulent insurance claims are sometimes filed.When a breeding ram is insured fraudulently, its identity cannot be verified, making it difficult to carry out sheep group insurance. Therefore, precise identification of individual sheep is necessary. To make the individual sheep in the process of raising efficient disease prevention and control, livestock insurance and accurate feeding, their individual identity identification has become a difficult point in the modern livestock industry towards intelligent pastures. In addition, livestock farming occupies an important position in the agricultural economy, of which sheep farming is an important part of the livestock industry. Moreover, sheep individual identification has become a common problem for individual identification of livestock, and its individual identification has become a prerequisite for achieving behavior monitoring^3–5^, precision feeding^6–8^, disease prevention^9–11^, and control^12–14^, food traceability, and individual management^15,16^, which are inevitable requirements for achieving intelligent livestock and poultry production.

Deep learning has emerged as a prominent topic in the fields of machine learning and pattern recognition, exhibiting remarkable efficiency in numerous image classification tasks.Deep learning learns to classify images, text and even sounds by example and can outperform humans in most application tasks. In addition, Convolutional Neural Networks (CNN) is considered to be the most popular and effective deep learning method in all previous scientific literature. Many popular CNNs are used to recognize images directly and it is becoming very common that although these networks can achieve good recognition results on benchmark dataset, the inconsistency of the problems to be solved in different real-world tasks can make the recognition results deviate significantly when using a specific CNNs. Therefore, it is necessary to design network models to solve specific problems. For example, changing the structure of the network, adding residual structure to the network, adding attention to the features, and other architectures.

Currently, there is a dearth of research in the area of individual sheep face recognition. This is primarily due to several challenges, such as the low autonomous mobility of individual sheep, the difficulty of biological image acquisition, the environmental influence that makes the animal’s face produce tear stains, mud and other factors that weaken its face biometric features. Moreover, the biometric features of individual sheep are far more complex than those of human beings, making it impractical to directly apply existing face recognition technologies to individual sheep recognition. However, due to the strong correlation between human faces and individual sheep faces, scholars and researchers worldwide are actively exploring and researching in this field. In sheep individual identity identification, the identification methods are mainly divided into two types, invasive and non-invasive. The invasive-based traditional livestock identification methods are broadly divided into three categories, Permanent Animal Identification Methodology (PIM), Semipermanent Animal Identification Methodology (SIM) and Temporary Identification Methodology (TIM)^17,18^. Permanent identification methods include methods such as ear notching^19^, ear grooves^20^, and skin branding^21^. Semi-permanent identification methods usually use neck chains and ear tags to mark animals for identification, and temporary identification methods use methods such as radio frequency identification (RFID) with hair spray paint to mark livestock for identification. Traditional identification methods, such as ear notching, tattooing and branding, are not reliable enough and can be easily changed and copied. Most agricultural countries use RFID technology embedded in ear tags for livestock applications. RFID ear tags are inexpensive, easy to identify, and have made some progress in animal identification. However, Johnston^22^ and Wardrope^23^ pointed out that the long-term use of ear tags can be dislodged, damaged, forged, and copied, which reduces the accuracy and credibility of individual sheep identification. The above-mentioned traditional sheep individual identification techniques cannot reliably identify individuals and also cause individual sheep stress when installing the equipment, which makes the animal welfare not satisfied and is no longer suitable for large-scale use in modern pastures. Non-intrusive sheep individual identification mainly uses the unique and stable biometric features of the sheep’s face, which contains a wealth of local and global features and is the most intuitive and visible external visual information that is unique and cannot be imitated.

In individual recognition based on individual sheep faces, Yang et al.^24^ proposed a triple interpolation feature method for face and sheep face calibration, which was used for training on sheep face dataset and obtained good results. In 2017, Lu et al.^25^ at the University of Cambridge, UK, achieved sheep pain level by collecting sheep face image information and processing with image calibration and feature extraction for automatic estimation of pain level of sheep, using histogram and support vector machine, and this experiment provided a method for extracting sheep face features. szymanski et al.^26^ investigated the feasibility of deep learning to assign kinship in livestock for genetic assessment by creating two CNN models, one method was used for face detection with a test accuracy of 80%. Salama et al.^27^ proposed a sheep face recognition method combining deep learning (DL) and Bayesian optimization, which automatically adjusted the convolutional neural network using Bayesian algorithm with recognition accuracy up to 98%.However, their approach solely involved modifying the neural network parameters, without effectively leveraging the feature information present in sheep faces. Xue^28^ proposed a Dlib-based sheep face key point detection with center metric for sheep face recognition method, which achieves sheep face recognition by measuring the feature distance of different sheep faces, and achieves better recognition results.Although facial recognition methods for individual sheep offer non-invasive and cost-effective data acquisition, and easy operation, existing studies in this field have mainly focused on proposing methods for feature extraction or classification without considering the impact of environmental factors on key facial features of sheep. This limitation has resulted in reduced accuracy in sheep face classification tasks. Li et al.^29,30^ to enable the deployment of sheep face recognition on edge devices, the authors enhanced the extraction of fine-grained sheep facial features while suppressing background interference by adding a Transformer module to the MobileViTV2 model. They achieved recognition accuracies of 96.94% and 97.13% on dataset containing 105 sheep with 5490 sheep face images and 186 sheep with 7434 sheep face images, respectively. Zhang et al.^31^ in the context of sheep face recognition tasks, an optimized Vision Transformer (ViT) model was developed. This was achieved by incorporating the LayerScale module into the encoder of the ViT-Base-16 model and utilizing transfer learning to enhance recognition accuracy. The model achieved an impressive recognition accuracy of 97.9% on a dataset consisting of 160 sheep facial images. Meng et al.^32^ to accomplish the task of sheep face recognition, a two-stage method was employed. Firstly, an object detection network was used to detect sheep faces in videos and crop these sheep face images. Subsequently, the cropped sheep face images were fed into EfficientNet and ResNet for feature encoding, enabling sheep face recognition based on sheep identity IDs. Experiments were conducted on a dataset comprising 547 sheep, resulting in an 85% recognition accuracy for sheep identification. Yang et al.^33^ proposed a lightweight sheep face detection model based on RetinaFace, designed for sheep face and facial landmark detection tasks. Although this model focuses primarily on sheep face detection and key point detection tasks, it lays the foundation for feature extraction and edge deployment in the context of sheep face recognition tasks. Su et al.^34^ applied the YOLOv4 model and further introduced the Convolutional Block Attention Module (CBAM) to enhance the model’s feature extraction capability. They conducted sheep recognition experiments on two different groups of 67 one- to two-year-old small-tailed Han sheep, achieving recognition accuracies of 91.58% and 90.61%, respectively. Therefore, an attention residual module is experimentally designed to enhance the feature description extracted by the CNN. The Attention Residual Module can fully utilize the local and global features of two adjacent feature layers of the backbone feature network, enhancing the performance of the feature extraction network and thus improves the recognition accuracy of this specific target of sheep faces.

## Materials and methods

### Overall framework

**Figure 1:**
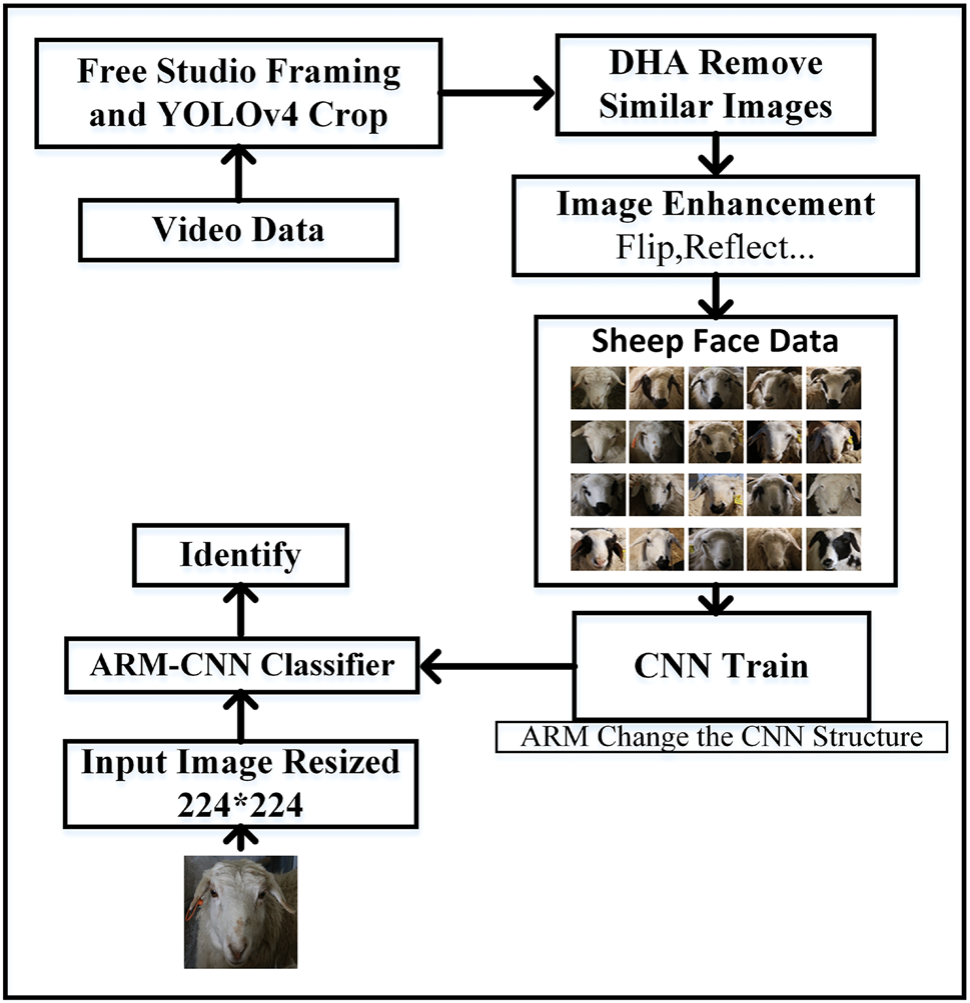
General flow of sheep face recognition.

The overall sheep face recognition method is shown in Fig. 1. The proposed approach for individual sheep face recognition involves the use of a spherical camera to capture image data, followed by frame splitting and sheep face interception from video data. Subsequently, image de-duplication and enhancement are applied to the intercepted sheep faces to create a dedicated dataset for sheep face recognition. Finally, the network is trained using the customized dataset to achieve accurate recognition of individual sheep faces.

### Dataset

**Figure 2:**
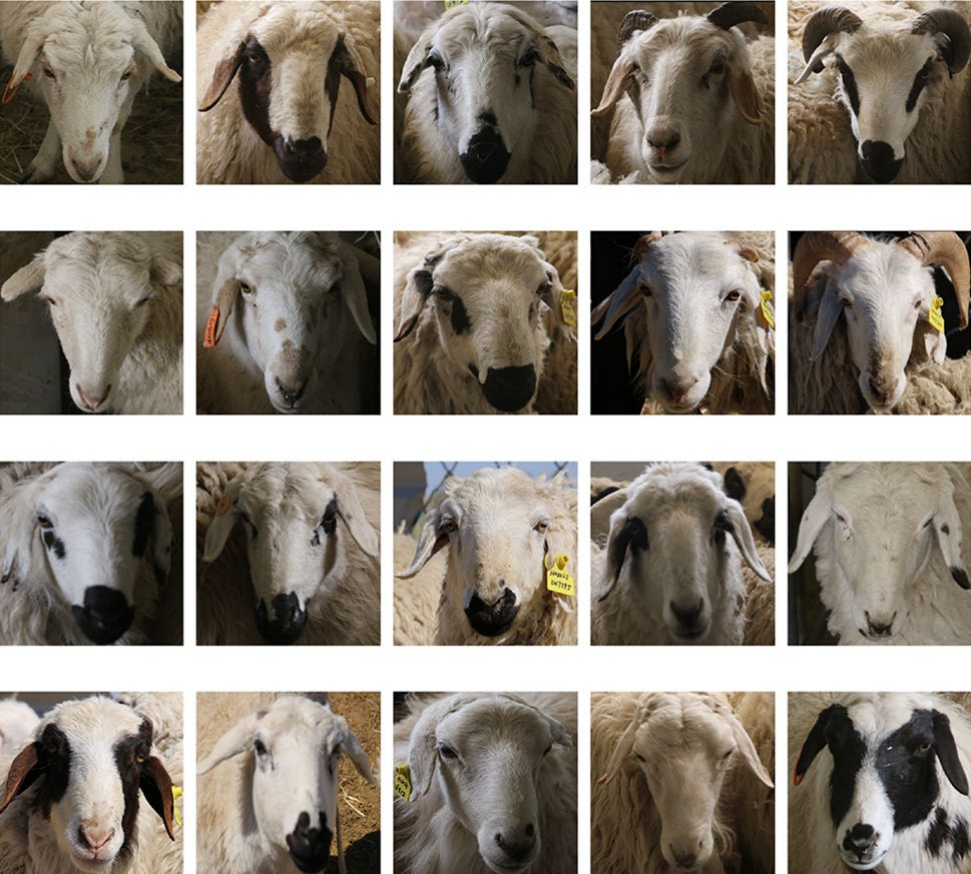
Sheep face dataset.

The experimental data were collected from the sheep breeding base in the Hailiutu Science and Technology Park of Inner Mongolia Agricultural University. The age of individual sheep ranged from 2 to 5 years old and had different physiological functions. To prevent the influence of light and environment on the image data quality at different time periods, the individual sheep video data were collected from 8:00 am to 12:00 pm daily, and the data were collected over a period of four months. The video data were captured by multiple dome cameras, and finally 133 videos of 38 individual sheep were collected, with each sheep having approximately 3–4 segments of 10–20 min each. Free Studio software was used to split the frames of the acquired individual sheep videos, and one individual sheep image was output every 30 frames, for a total of 1500 images per sheep, and a total of 57000 images for 38 sheep. Additional processing of the framed images is necessary due to the presence of extensive background and invalid frames in individual sheep images after framing. Therefore, it is necessary to remove the similar images from the cropped images. The experiment uses the Difference Hash Algorithm (DHA) to detect the similarity of the images. Firstly, the detail components and high frequency components of the sheep face images are hidden, the images are resized to 9x8 size and converted to grayscale images. Secondly, the intensity of adjacent pixels in the grayscale images is calculated by the gradient principle to derive the difference value, the difference value is calculated separately for each row, and there are 9 pixels in each row. The difference value is calculated separately for each line, there are 9 pixels in each line, through the calculation will get 8 difference values, a total of 64 difference values of a picture, and then calculate the Hamming distance of the difference values of the two pictures, and finally remove one of the two pictures in which the Hamming distance is less than 5. Finally, the invalid frames of sheep face data due to lighting changes and motion blur are manually removed. After removing the invalid frames and similar images, a total of 4490 images of 38 individual sheep were obtained, and the image data of part of the sheep face dataset is shown in Fig. 2.

**Figure 3:**
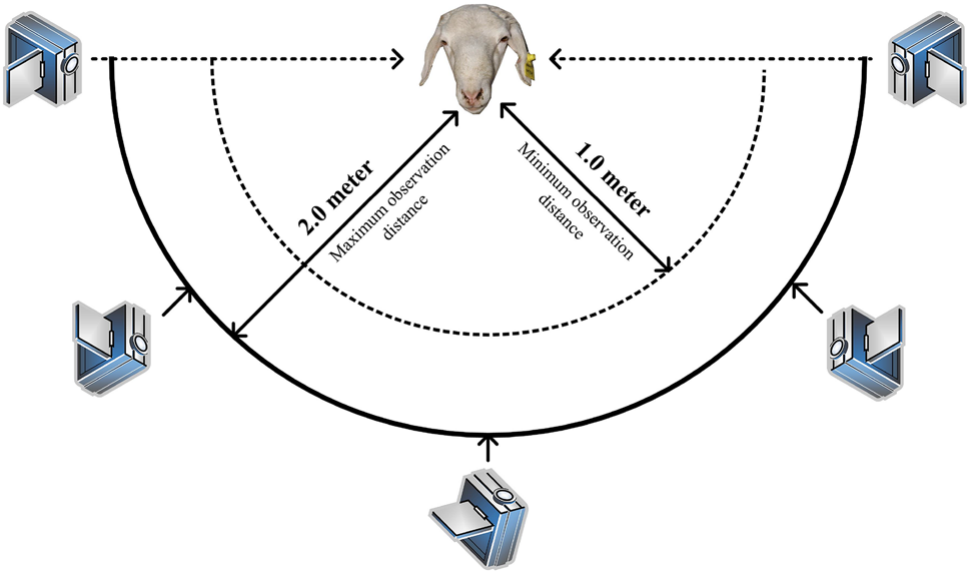
Schematic diagram of individual video capture of sheep.

Figure 3 illustrates the acquisition process of individual sheep face data, the spherical camera facing the individual face of the sheep at different angles and parts of the distance interval between 1–2 m. In order to collect the sheep face at different angles, the spherical camera makes angle adjustment every 20 s.

### Data augmentation

**Figure 4:**
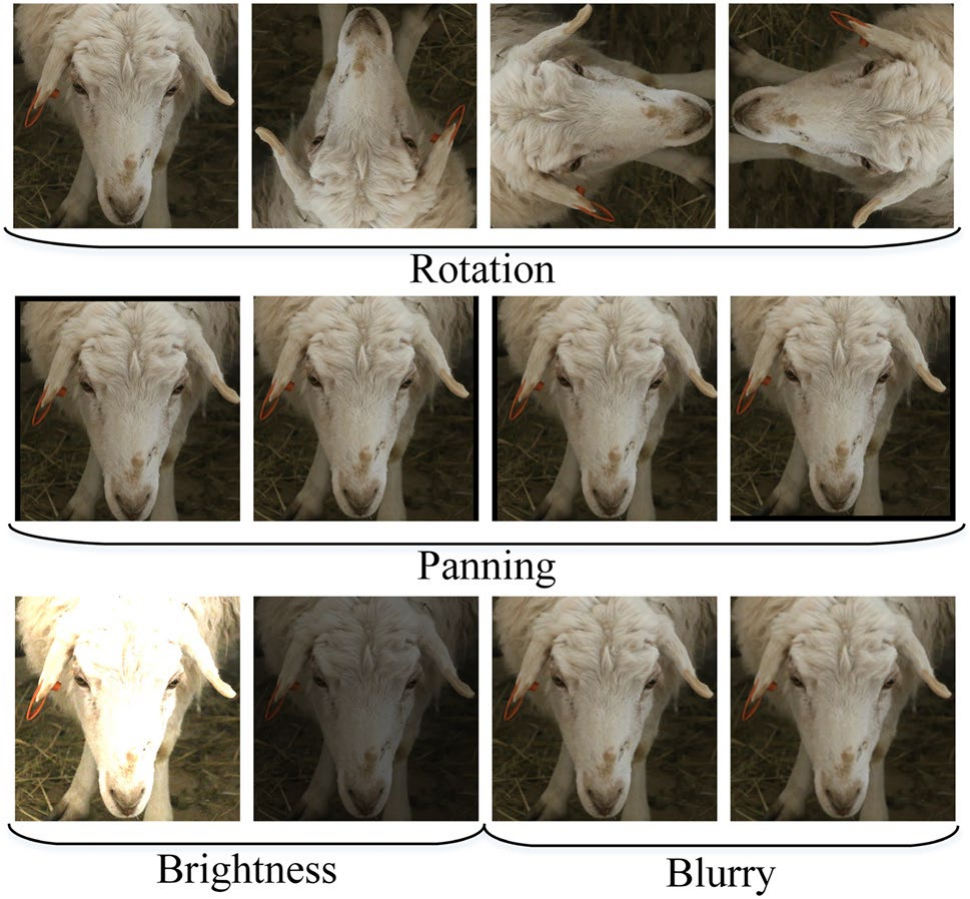
Partial data enhancement of sheep face image data.

DL models have a high parameter count, and in order to achieve good image recognition results using these models, a large amount of training data is necessary to train the neural network’s weight parameters and improve network performance. Because the experiment uses a custom sheep face recognition dataset, there is a shortage for the amount of sheep face image data. Therefore, the experiments will use image enhancement techniques to derive new images from the existing sheep face image data, so as to supplement the number of training images and prevent the overfitting problem caused by the insufficient amount of sheep face image data. To achieve better control over the effect of data augmentation, this study employs an offline approach to increase the quantity of sheep face image data. Specifically, the sheep face image data is augmented and images are generated prior to model training.We use horizontal mirroring, panning,brightness adjustment and blurring for image enhancement. After the image enhancement operation, the amount of sheep face image data is expanded by 11 times. The enhanced sheep face data is shown in Fig. 4.

### Method

**Figure 5:**
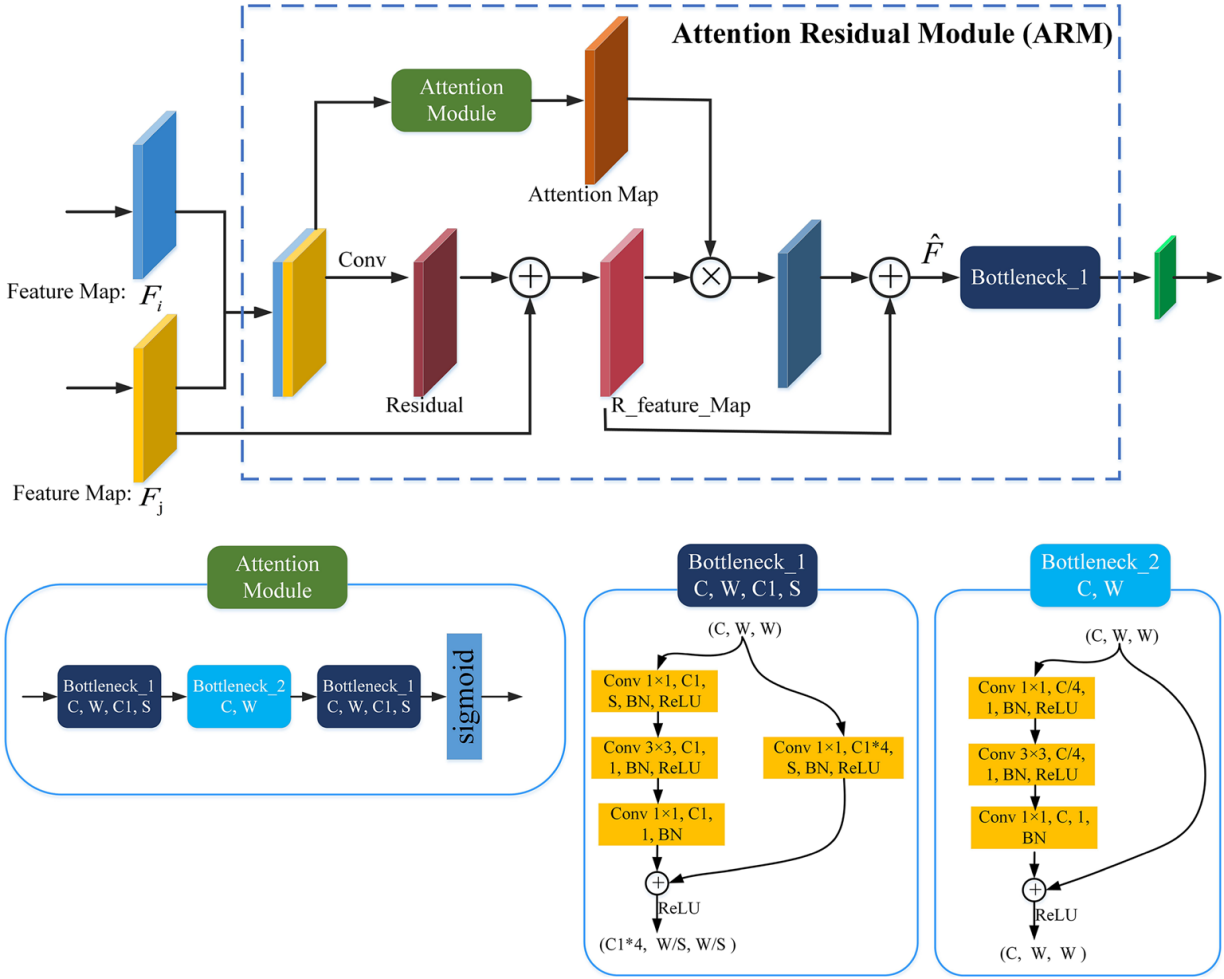
Structure of attentional residual fusion module.

The deeper layers of CNN can extract features with larger perceptual fields and richer semantic information, which enhances the robustness of the model to pose changes, occlusions, and local deformations in individual sheep faces. However, the lower resolution of the deeper layers may result in the loss of geometric detail information in sheep faces. In contrast, the shallow network is able to extract local feature information such as texture and edge of the image during training. Although the feature map resolution of the shallow network is high, the fixed size of the convolutional kernel makes the semantic representation of features poor. With the gradual deepening of the network layers, the recognition performance of the network also improves and then decreases with the increase of the perceptual field, which shows that the appropriate contextual information helps to improve the recognition ability. According to the above problem, to make full use of the local and global features of different feature layers in the CNN an Attention Residual Module (ARM) is experimentally designed to make full use of the features. ARM is designed to fully utilize the local and global features of two adjacent feature layers of the feature extraction network to improve the performance of the CNN. The ARM structure is shown in Fig. 5.

As shown in the above figure, the ARM first splices the As shown in the above figure, the ARM first splices the feature maps obtained from two feature layers and then passes them into both the attention module and the residual structure. The residual structure learns complementary feature information in different feature maps while retaining the original features, and R_feature_Map is the output of the detailed features generated by the residual module. The attention module generates an attention feature map by learning, and then multiplies R_feature_Map with the attention feature map. The attention feature map acts as a feature selector, which can effectively enhance good features and suppress useless features in R_feature_Map. Finally, the R_feature_Map is added element by element with the enhanced feature map, and the output feature map is obtained by adjusting the size of the feature map through a residual structure with a convolution step of 2. The formula for calculating the ARM feature map is shown in Eq. (1).

1 $$\begin{aligned} \hat{F}=\left\{ 1+{\text {Att}}\left[ {\text {concat}}\left( F_i, F_j\right) \right] \right\} \times \left\{ \Phi \left[ {\text {concat}}\left( F_i, F_j\right) \right] +F_j\right\} \end{aligned}$$

where $F_i$ and $F_j$ are the input feature maps in the ARM, respectively, $\hat{F}$ is the unscaled feature map, ${\text {concat}}\left( F_i, F_j\right)$ represents the splicing operation of $F_i$ and $F_j$, ${\text {Att}}\left[ {\text {concat}}\left( F_i, F_j\right) \right]$ represents the feature map learned by the attention module, and $\Phi$ represents the residual function.

2 $$\begin{aligned} {\text {att}}(C, x, y)=\frac{1}{1+\exp [-D(C, x, y)]} \end{aligned}$$

where ${\text {att}}(C, x, y)$ denotes the weight learned at the *C*channel (*x*, *y*) position and *D*(*C*, *x*, *y*) denotes the feature value at the *C* channel (*x*, *y*) position.

**Figure 6:**
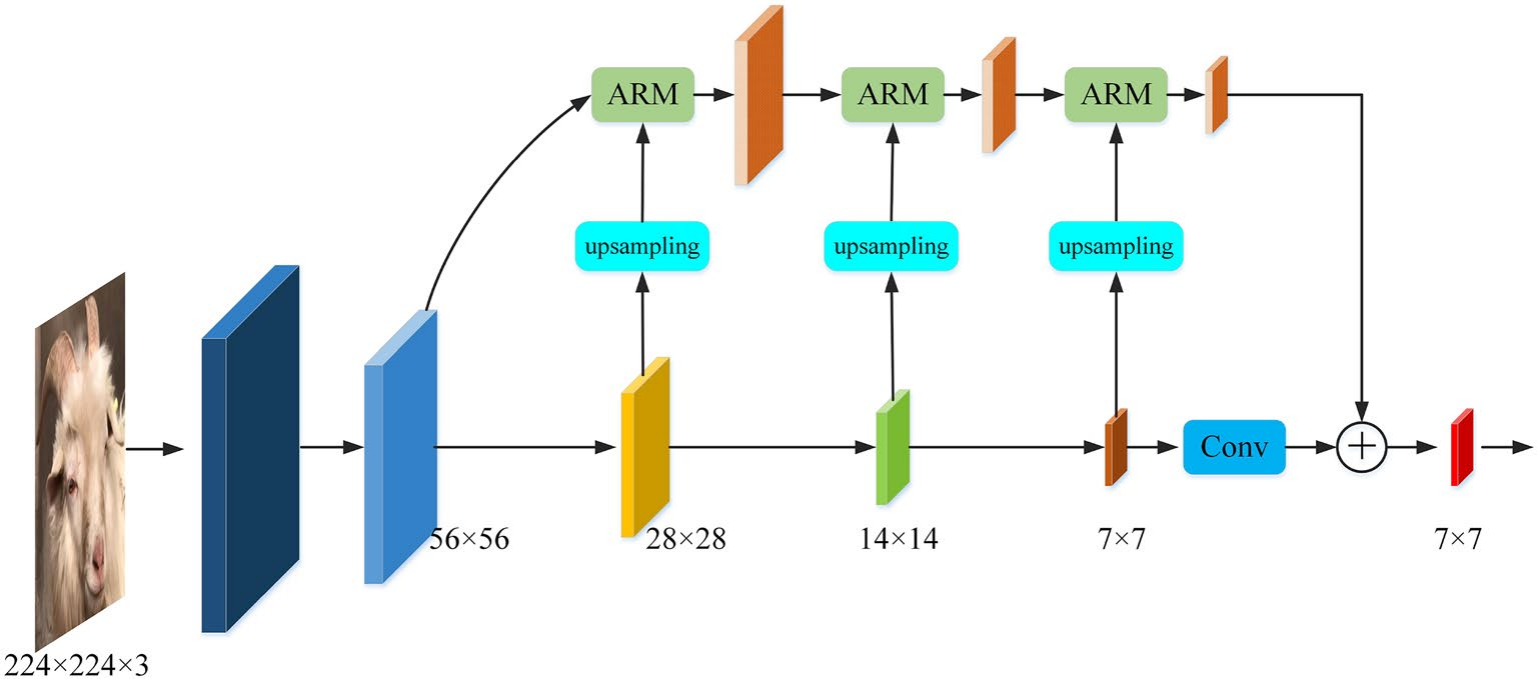
ARM feature aggregation process.

To investigate the effect of the ARM on the accuracy of sheep face recognition task, the experiment will use VGG16^35^, GoogLeNet^36^ and ResNet50^37^as the feature extraction network for sheep face recognition, because the input image size of the three models is equal, so during the training process VGG16, GoogLeNet and ResNet50 can all obtain $56\times 56$, $28\times 28$, $14\times 14$, and $7\times 7$ feature maps. In order to fully utilize the features of each feature layer, the $28\times 28$ feature map is first upsampled and passed into the ARM at the same time as the feature map of the previous layer to integrate the spatial information, and then the deeper feature map is upsampled and integrated with the feature map obtained from the previous layer, and finally the feature map of the last layer of the feature extraction network is integrated with the ARM. Finally, the last layer of the feature extraction network and the integrated feature map of the ARM are summed pixel by pixel to obtain the final feature output. The feature integration process is shown in Fig. 6.

### Peer review

*Nature Communications* thanks the anonymous reviewer(s) for their contribution to the peer review of this work.

## Results and discussion

### Experimental equipment

The software platform selected for the sheep face recognition experiment is Anaconda3, using the Tensorflow framework for feature extraction network construction, processor is intelCorei5_9300 CPU@2.4GHz RAM size is 16G, 64-bit operating system, graphics card model is NVIDIAGeForce GTX1660Ti, video memory size is 6G.

### Training strategy of sheep recognition model

In this section, we discuss the training strategy employed for the sheep recognition model. We utilized benchmark networks, namely VGG16, GoogLeNet, and ResNet50, as a basis for sheep face recognition to assess the impact of ARM on the task.

The experiment’s objective is to compare the performance of various CNNs, including VGG16, GoogLeNet, and ResNet50, as well as ARM-enhanced versions (ARM_VGG16, ARM_GoogLeNet, and ARM_ResNet50), regarding the accuracy of sheep face recognition. This evaluation was conducted using a custom dataset, which was divided into training, validation, and test sets with proportions of 70%, 15%, and 15%, respectively.

The images used for network training were resized to 224x224x3 pixels and normalized before input into the CNN. This preprocessing step helps prevent gradient explosions during the training process, ensuring model stability. The network underwent a total of 200 training epochs using transfer learning, with a batch size of 16. The chosen optimizer was Adam, with a learning rate set to 0.0001.

**Figure 7:**
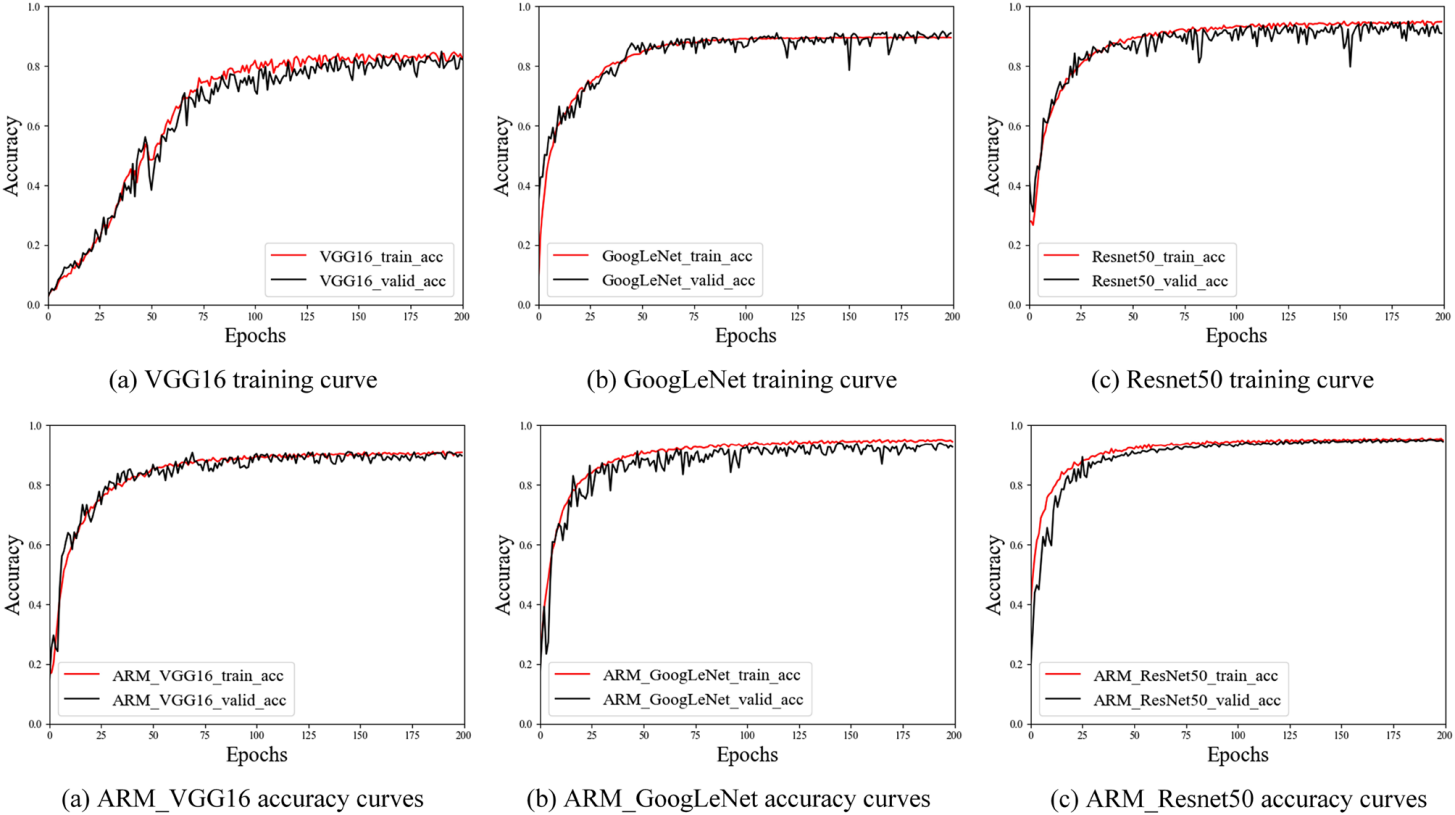
Model training set and validation set accuracy curves.

Real-time accuracy monitoring during network training was achieved using Tensorboard. Figure 7 illustrates the accuracy changes on both the training and validation sets throughout the training process.

From the above Fig. 7, it can be seen that the training accuracy of VGG16 is more jittery and less accurate in the training and validation sets; the accuracy of GoogLeNet is smooth in the training set, and the accuracy jitters in the validation set are relatively calm compared with VGG16 and ResNet50; ResNet50 is faster in learning speed compared with VGG16 and GoogLeNet, and its accuracy in both the training and validation sets of the custom sheep face recognition dataset is higher than that of VGG16 and GoogLeNet. The feature extraction network with ARM reduces the jitter between the training set and the test set compared with the feature extraction network without ARM, which improves the training accuracy of the network. Especially for the VGG16 model, the addition of the ARM makes the training of the VGG16 model easier and significantly improves the training accuracy of the model.

**Figure 8:**
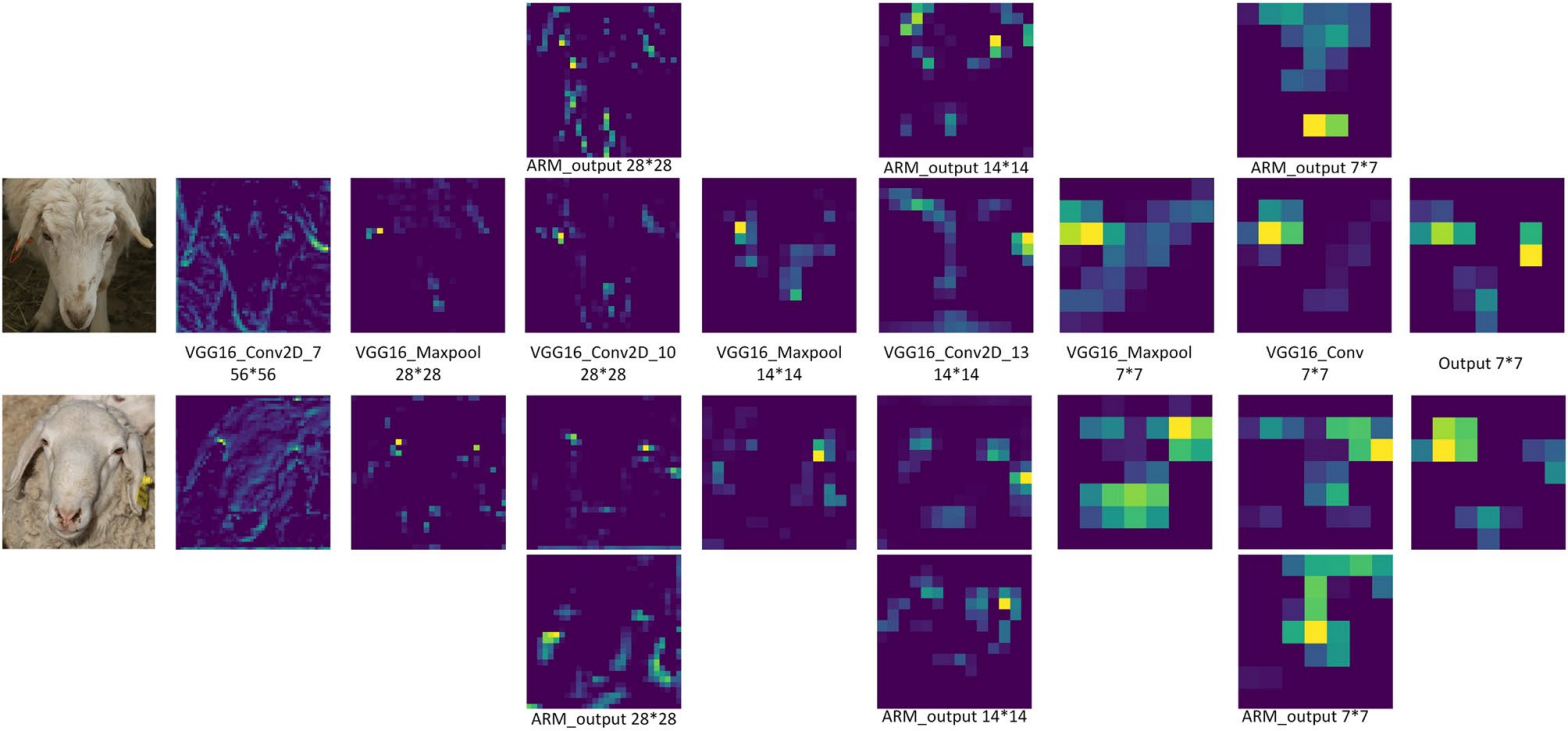
ARM_VGG16 network partial convolution layer, pooling layer for visualization.

The experiments visualized the output feature maps of some of the convolutional layers, pooling layers and ARM of the ARM_VGG16 network, aiming to better understand the feature aggregation characteristics of the ARM. The visualization is shown in Fig. 8.

The heat map of the sheep face reveals that the VGG16 network reduces the scale of the sheep face feature map by half with each layer through down-sampling operations. As a result, the network improves its ability to abstract the image and extract local information such as texture and edges from the shallow layers. Meanwhile, the deeper layers capture the global semantic information of the sheep face.By feeding both the deep and shallow feature maps into the ARM, the module is able to enhance the useful information while suppressing the useless information. This results in improved sensitivity of the network to the salient features of the sheep face, making the sheep face features more prominent. These findings demonstrate the effectiveness of the ARM in aggregating features across different layers, ultimately leading to improved accuracy in sheep face recognition.

### Test result of sheep recognition model

In order to get a comprehensive measure of the impact of the ARM on the performance of the convolutional neural network model, we examined the following five metrics on the test set: Precision, Recall, F1-score, Params, and Cost. the test results are shown in Table 1.

**Table 1:** Test set evaluation index.

Model	Precision%	Recall%	F1-score%	Parms	Cost (s)
VGG16	82.26	85.38	83.79	$8.3\times 10^6$	0.0547
ARM_VGG16	92.46	93.43	92.94	$9.9\times 10^6$	0.0624
GoogLeNet	88.03	90.23	89.11	$15.2\times 10^6$	0.0621
ARM_GoogLeNet	94.68	93.28	93.97	$17.8\times 10^6$	0.0646
ResNet50	93.67	93.22	93.44	$16.7\times 10^6$	0.0456
ARM_ResNet50	98.05	98.38	98.21	$17.9\times 10^6$	0.0513
vision_transformer	95.33	94.26	94.79	$15.5\times 10^6$	0.0451
MobileVIT	93.38	92.55	92.96	$0.9\times 10^6$	0.0095

Precision: refer to the proportion of positive samples in positive examples determined by the classifier.

3 $$\begin{aligned} \text { Precision }=\frac{T P}{T P+F P} \end{aligned}$$

Recall: refer to the ratio of predicted positive cases to the total positive cases

4 $$\begin{aligned} \text { Recall }=\frac{T P}{T P+F N} \end{aligned}$$

F1-score: it is the harmonic average of precision rate and recall rate, with the maximum of 1 and the minimum of 0.

5 $$\begin{aligned} \text { F1-score }=2 \times \frac{\text { Precision } \times \text { Recall }}{\text { Precision }+\text { Recall }} \end{aligned}$$

Params is a standard to measure the amount of network model parameters. Cost is the time consumed to predict a picture.

6 $$\begin{aligned} {\text {Cos}} t=\frac{T_n}{n} \end{aligned}$$

TP (True Positive): predicts the answer correctly, FP (False Positive): wrongly predicts other samples as this category, FN (False Negative): this category label is predicted as other, Tn (time all): the time taken to predict n images.

From the test results, we can see that the recognition accuracy of the benchmark networks VGG16, GoogLeNet and ResNet50 reached 82.26%, 88.03% and 93.67%, respectively. When the ARM was not added to the benchmark networks, and the comparison of the three different benchmark networks proved that different convolutional neural networks have an impact on the specific task of sheep face recognition. When the benchmark networks were added to the ARM ARM_VGG16 model achieves 92.46% accuracy for sheep face recognition when the ARM is added to the benchmark network, which is a 10.2% improvement compared to the VGG16 model and the largest performance improvement among the three benchmark networks. The ARM_ResNet50 sheep face recognition accuracy is the highest and reaches 98.05%, and the sheep face recognition accuracy is improved by 4.38% compared to the ResNet50 model. Although there is a small increase in the number of parameters and detection time after adding the ARM, the improvement in accuracy is very significant, which also proves the effectiveness of the proposed ARM. Therefore, applying the ARM to the convolutional neural network can effectively aggregate the feature mapping between different layers of the neural network, which is beneficial to improve the accuracy and generalization ability of the network. From Table 1, it can be seen that our proposed ARM model achieves the highest Precision, Recall, and F1-score. Both vision_transformer and MobileVIT have fewer parameters compared to our ARM model, with MobileVIT having the fewest parameters at $0.9\times 10^6$. However, the accuracy of the MobileVIT model is the lowest, with a relative decrease of 4.67% compared to our proposed ARM model. Additionally, MobileVIT models are commonly used for deployment on edge devices in deep learning applications, where model lightweightness is a crucial consideration.However, for theoretical research in sheep face recognition tasks, we are more inclined to prioritize individual recognition accuracy.

### Comparison of attention models

**Figure 9:**
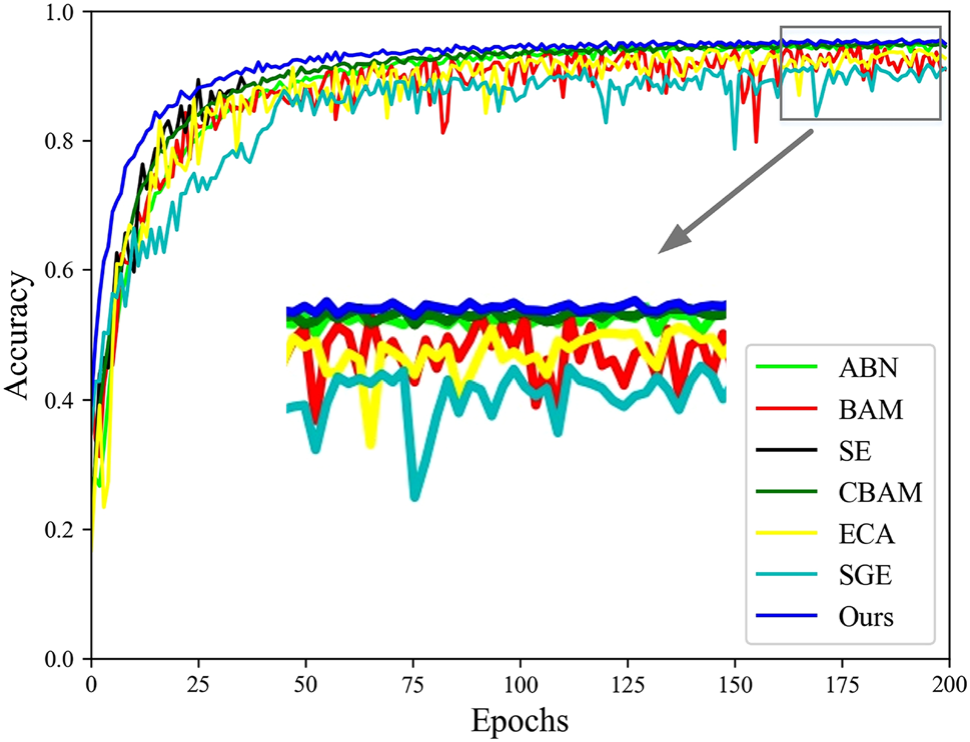
Training accuracy curves for attention mechanisms.

To validate the effectiveness of the ARM attention mechanism, the experiment compared it with six other attention mechanisms: ABN, BAM, CBAM, SE, ECA, and SGE. We selected the VGG model as the baseline network to assess the performance of these attention mechanisms. Additionally, k-fold cross-validation was applied during the training process to comprehensively evaluate the models’ generalization performance. We set the number of folds to 5 for training the models, selected the best hyperparameter configurations from the experiments, and trained each fold for 200 epochs. The Adam optimizer was used with a learning rate of 0.00001, and a batch size of 16 was employed. The training accuracy is shown in Fig. 9.

In Fig. 9, the training results show that ABN, BAM, CBAM, SE, ECA, and SGE, as well as our ARM model, continuously improve in accuracy during the training process. In the early stages of training, our proposed ARM attention module shows the fastest improvement in accuracy. ABN, CBAM, and SE approach the recognition accuracy of ARM in the later stages of training. However, it can be seen from the graph that our proposed ARM attention mechanism achieves the highest recognition accuracy for the specific task of sheep face recognition. ECA, BAM, and SGE attention mechanisms exhibit greater fluctuation in accuracy during training and perform poorly in the sheep face recognition task.

To comprehensively evaluate the impact of ABN, BAM, CBAM, SE, ECA, SGE, and ARM on convolutional neural network model performance, we will use the following five metrics on the test set, including Precision, Recall, F1-score, and Params. The test results for the attention models are shown in Table 2.

**Table 2:** Evaluation metrics on the test set for attention mechanisms.

Attention	Precision%	Recall%	F1-score%	Parms (MB)
ABN	97.89	98.33	98.10	515.25
BAM	95.46	94.35	94.90	512.28
SE	98.06	97.46	97.76	513.85
CBAM	97.96	98.03	97.99	512.24
ECA	93.53	93.22	93.37	512.26
SGE	93.03	93.64	93.33	512.24
Ours	98.05	98.38	98.21	405.26

From the test results, it can be seen that SE achieves the highest recognition accuracy at 98.06%. However, our proposed ARM module is only 0.01% lower in recognition accuracy compared to the highest. In the table, ARM has the best Recall, F1-score, and Params among these attention mechanisms, which also demonstrates the positive impact of applying the ARM module to convolutional neural networks for the specific task of sheep face recognition.

## Conclusion and future work

### Conclusion

Deep learning is a hot topic, and CNN is the most effective deep learning method for visual recognition problems. However, not all CNN models can accurately perform feature extraction for a specific task. Experimentally, an ARM is proposed to aggregate feature mapping between different feature layers of CNN for the specific problem of sheep face recognition, and the proposed method is tested on a custom sheep face recognition dataset by combining VGG16, GoogLeNet and ResNet50 benchmark networks. The results confirm the effectiveness of the ARM and also provide a basis for solving specific tasks in reality.

### Future work

The experiment conducted for sheep face recognition was limited to a single type of sheep, and the size of the sheep face dataset used was small. Therefore, there is a need to expand the dataset further to improve the recognition accuracy. Moreover, the recognition of individual sheep belonging to different breeds or types would require additional investigation.

The experimentally constructed sheep face dataset had limited diversity, as it only contained images of a single type of sheep, and therefore, the recognition of different kinds of individual sheep needs to be further investigated. Additionally, individual sheep faces may exhibit changes in pose, expression, and lighting, which can affect the accuracy of recognition. Therefore, future research should address these challenges. Furthermore, the deep learning model used in this study had a large number of parameters, which may hinder its deployment on edge devices. To address this issue, future work could explore methods such as knowledge distillation and pruning to make the sheep face recognition model more lightweight and suitable for deployment on edge devices.

## Data Availability

The data that support the findings of this study are available from the corresponding author upon request.

## Code availability

The codes used for the simulations are available in https://github.com/zsdragons/sheep_face.

## References

[1] Schommer TJ, Woolever MM (2008). A Review of Disease Related Conflicts Between Domestic Sheep and goats and Bighorn Sheep.

[2] Nikolov D, Anastasova-Chopeva M, Ivanova E (2013). Insurance products in agriculture and farm insurance behaviour. Econ. Thought J..

[3] Nóbrega, L., Tavares, A., Cardoso, A. & Gonçalves, P. Animal monitoring based on iot technologies. In *2018 IoT Vertical and Topical Summit on Agriculture-Tuscany (IOT Tuscany)* 1–5 (IEEE, 2018).

[4] Wang, Y. et al. The design of an intelligent livestock production monitoring and management system. In *2018 IEEE 7th Data Driven Control and Learning Systems Conference (DDCLS)* 944–948 (IEEE, 2018).

[5] Alonso RS, Sittón-Candanedo I, García Ó, Prieto J, Rodríguez-González S (2020). An intelligent edge-IoT platform for monitoring livestock and crops in a dairy farming scenario. Ad Hoc Netw..

[6] Memon, M. H. et al. Internet of Things (IoT) enabled smart animal farm. In *2016 3rd International Conference on Computing for Sustainable Global Development (INDIACom)* 2067–2072 (IEEE, 2016).

[7] Mertens, K., Decuypere, E., De Baerdemaeker, J. & De Ketelaere, B. Intelligent monitoring of livestock production processes based on synergistic control. In *2010 Pittsburgh, Pennsylvania, June 20–June 23, 2010* 1 (American Society of Agricultural and Biological Engineers, 2010).

[8] Zurita-Herrera P, Delgado Bermejo JV, Argüello Henríquez A, Camacho Vallejo ME, Germano Costa R (2013). Effects of three management systems on meat quality of dairy breed goat kids. J. Appl. Anim. Res..

[9] Shinde TA, Prasad JR (2017). IoT based animal health monitoring with Naive Bayes classification. Int. J. Emerg. Trends Technol..

[10] Lianou DT (2020). A detailed questionnaire for the evaluation of health management in dairy sheep and goats. Animals.

[11] Solaiman SG (2010). Goat Science and Production.

[12] Velthuis A (2009). Costs and reliability of livestock traceability systems for the Dutch sheep and goat sectors. Acta Agric. Scand. Sect. C.

[13] Yan C (2018). Traceability information modeling and system implementation in Chinese domestic sheep meat supply chains. J. Food Process. Eng..

[14] Stanford K, Stitt J, Kellar J, McAllister T (2001). Traceability in cattle and small ruminants in Canada. Rev. Sci. Tech. Off. Int. Epizoot..

[15] Li, L. et al. Research on goat health management system. In *2011 3rd International Workshop on Intelligent Systems and Applications* 1–4 (IEEE, 2011).

[16] Lianou DT, Fthenakis GC (2021). Dairy sheep and goat farmers: Socio-demographic characteristics and their associations with health management and performance on farms. Land.

[17] Wang, Z., Su, Y., Yang, F. & Zhang, X. Application of radio frequency identification (rfid) technology in goat dairy traceability. In *2017 7th International Conference on Education, Management, Computer and Society (EMCS 2017)* 76–80 (Atlantis Press, 2017).

[18] Roberts CM (2006). Radio frequency identification (rfid). Comput. Secur..

[19] Leslie E, Hernández-Jover M, Newman R, Holyoake P (2010). Assessment of acute pain experienced by piglets from ear tagging, ear notching and intraperitoneal injectable transponders. Appl. Anim. Behav. Sci..

[20] Jun-Xian, L. U. et al. The identification of pig origin ingredients in livestock and poultry meat based on fluorogenic quantitative pcr. Food Research and Development (2017).

[21] Lay D, Friend T, Bowers C, Grissom K, Jenkins O (1992). A comparative physiological and behavioral study of freeze and hot-iron branding using dairy cows. J. Anim. Sci..

[22] Johnston A, Edwards D (1996). Welfare implications of identification of cattle by ear tags. Vet. Rec..

[23] Wardrope D (1995). Problems with the use of ear tags in cattle. Vet. Rec..

[24] Yang, H., Zhang, R. & Robinson, P. Human and sheep facial landmarks localisation by triplet interpolated features. In *2016 IEEE Winter Conference on Applications of Computer Vision (WACV)* 1–8 (IEEE, 2016).

[25] Lu, Y., Mahmoud, M. & Robinson, P. Estimating sheep pain level using facial action unit detection. In *2017 12th IEEE International Conference on Automatic Face & Gesture Recognition (FG 2017)* 394–399 (IEEE, 2017).

[26] Szymanski, L. & Lee, M. Deep sheep: Kinship assignment in livestock from facial images. In *2020 35th International Conference on Image and Vision Computing New Zealand (IVCNZ)* 1–6 (IEEE, 2020).

[27] Salama A, Hassanien AE, Fahmy A (2019). Sheep identification using a hybrid deep learning and Bayesian optimization approach. IEEE Access.

[28] Xue H (2021). Open set sheep face recognition based on Euclidean space metric. Math. Probl. Eng..

[29] Li X, Du J, Yang J, Li S (2022). When mobilenetv2 meets transformer: A balanced sheep face recognition model. Agriculture.

[30] Li X, Xiang Y, Li S (2023). Combining convolutional and vision transformer structures for sheep face recognition. Comput. Electron. Agric..

[31] Zhang X, Xuan C, Ma Y, Su H (2023). A high-precision facial recognition method for small-tailed Han sheep based on an optimized vision transformer. Animal.

[32] Meng, X., Tao, P., Han, L. & CaiRang, D. Sheep identification with distance balance in two stages deep learning. In *2022 IEEE 6th Information Technology and Mechatronics Engineering Conference (ITOEC)*, Vol. 6, 1308–1313 (IEEE, 2022).

[33] Yang, J. & Li, S. Research on lightweight sheep face detection model based on retinaface. In *2022 IEEE 10th Joint International Information Technology and Artificial Intelligence Conference (ITAIC)*, Vol. 10, 2390–2394 (IEEE, 2022).

[34] Zhang X, Xuan C, Ma Y, Su H, Zhang M (2022). Biometric facial identification using attention module optimized yolov4 for sheep. Comput. Electron. Agric..

[35] Simonyan, K. & Zisserman, A. Very deep convolutional networks for large-scale image recognition. arXiv:1409.1556 (2014).

[36] Szegedy, C. et al. Going deeper with convolutions. In *Proceedings of the IEEE Conference on Computer Vision and Pattern Recognition* 1–9 (2015).

[37] He, K., Zhang, X., Ren, S. & Sun, J. Deep residual learning for image recognition. In *Proceedings of the IEEE Conference on Computer Vision and Pattern Recognition* 770–778 (2016).

